# It is More Anxiousness than Role-playing: Social Camouflaging Conceptualization Among Adults on the Autism Spectrum Compared to Persons with Social Anxiety Disorder

**DOI:** 10.1007/s10803-024-06416-0

**Published:** 2024-06-06

**Authors:** Anna Pyszkowska

**Affiliations:** University of Silesia in Katowice, Grażyńskiego 53, Katowice, 40-007 Poland

**Keywords:** Camouflaging, Autistic burnout, Public stigma, Social anxiety, Autism

## Abstract

Purpose. Autistic individuals consider social camouflaging, e.g., masking autistic traits or social skills compensation, as exhausting and effortful, often leading to diminished well-being or burnout, as well as adaptive for satisfying social interactions. Developing camouflaging may result in isolation, social avoidance, increased self-stigmatization, and misdiagnosis, including social anxiety disorder. The study’s objective was to explore and conceptualize social camouflaging, with a particular focus on social anxiety symptoms, autistic burnout, and public stigma, among autistic individuals, with two comparative samples: with social anxiety disorder (SAD) and dual diagnoses (SAD + ASD). Methods. 254 individuals participated in the study (including 186 females, 148 with ASD diagnosis). CAT-Q, AQ-10, AASPIRE’s Autistic Burnout Scale, LSAS-SR, The Perceived Public Stigma Scale were used. Results. The findings suggest differences in the interrelation dynamics between the samples studied, with autistic burnout and social anxiety symptoms of essential significance in camouflaging strategies, and autistic traits being of secondary importance. Structural equation models showed that the proposed conceptualization, with camouflaging and autistic burnout as the outcome variables, exhibited acceptable fit, implying that this strategy is costly and may result in exhaustion. Conclusion. The total score of camouflaging did not differ between the groups studied, suggesting that a tendency to camouflage is rather transdiagnostic, deriving from anxiousness and negative self-perception, not being autistic per se.

## Introduction

Autism spectrum disorder (ASD) is considered a heterogeneous phenomenon, and it has been suggested that it might share a common etiopathological root with other psychiatric disorders and be the risk factor for developing mental health issues (Dell’Osso et al., 2019). Despite several studies proving that autistic traits are associated with higher anxiety, stress, (Hull et al., 2021b), suicidal ideations, and trauma-related experiences, these are not caused directly by autistic features (e.g., limited social skills, sensory sensitivity, and diminished executive functions) but are a reaction to the lack of ability to adapt to a non-autistic, often maladjusted, environment (Raymaker et al., 2020). In turn, the autism spectrum is associated with an increased prevalence of psychiatric disorders (Rosen et al., 2018), with overlapping diagnoses of 70% in this population. Therefore, a differentiation diagnosis between ASD and other disorders is challenging and yet to be fully understood, resulting in misdiagnoses, such as personality disorders (Iversen & Kildahl, 2022), social anxiety (Gesi et al., 2021), psychotic disorders (Demetriou et al., 2020), or delayed diagnosis in adulthood. Females are more likely to receive a delayed diagnosis or a misdiagnosis, with an average time lapse between first contact with mental health care and proper ASD diagnosis being eight-ten years (Gesi et al., 2021).

One of the potential reasons for a misdiagnosis is social camouflaging. The current literature depicts camouflaging in various behavioral contexts, characterizing it as a combination of (1) masking of autistic traits (e.g., refraining from self-stimulation), (2) social skills compensation (e.g., employing scripts for different social scenarios), and (3) assimilation to social situations (e.g., forcing oneself to develop a small-talk) (Hull et al., 2017). Livingston and Happe (2017) point out that compensation may be exhibited in deep or shallow form. The former is based on one’s executive functions and is more intuitive (e.g., based on one’s good memory), when the latter is more rigid and unintuitive (e.g., mimicking others’ behaviors without understanding the context). Research implies that females more often develop camouflaging, obtaining this strategy across more situations and for more of the time than males (Cassidy et al., 2018), affecting the diagnosis ratio. However, it does not mean that fewer females meet the diagnostic criteria; instead, they may elude traditional diagnostic methods that do not consider camouflage (Rynkiewicz et al., 2016).

Autistic individuals consider camouflaging as exhausting and effortful, often leading to diminished well-being (Cook et al., 2021) or burnout (Hull et al., 2017; Raymaker et al., 2020), and adaptive for developing satisfying social interactions (Miller et al., 2021; Bradley et al., 2021). A recurrent theme in camouflaging-related reports is inauthenticity, described as performing or playing a role (Livingston et al., 2019), which often results in the inability to meet (or acknowledge) other autistic people and, in turn, disallows for normalization of one’s experiences (Cook et al., 2022). In sum, camouflaging’s impact on well-being and self-perception depends on various contexts and motivations (Hull et al., 2021b). It is rather dialectic than dichotomic: persons on the spectrum implement strategies to navigate social contexts and blend into the environment, also mask their autistic traits due to stigma experiences or fear of exclusion (Petrolini et al., 2023). Researchers highlight that as the autistic spectrum is broad and heterogeneous, so is camouflaging. Therefore, not all autistic individuals experience camouflaging as unfavorable, e.g., those who face more visible autistic traits and thus are not in the position to hide or engage in deep compensation moves (Petrolini et al., 2023).

Perry et al. (2021) propose interpreting camouflaging through the lens of the “passing” phenomenon. Passing is a sociology-based construct referring to a person’s ability to be regarded as a member of an identity group or category different from their own (Goffman, 1963). A motivation to “pass” as non-autistic may derive from negative experiences from the past, acceptance seeking, or internalized stigma (treating negative stereotypes about autism as truth and incorporating them as beliefs about oneself). Given that persons on the autism spectrum often present self-stigmatizing beliefs (e.g., “Being autistic means I am worse than others”), camouflaging would be a consequence of negative self-perception and act as its behavioral manifestation (e.g., “I should be less autistic to be liked/accepted” would lead to camouflaging). Perry et al. (2021) propose considering camouflaging in this population via a Social Identity Framework (SIT; Tajfel & Turner, 2004), which suggests that group members seek to regain a positive identity through individualistic and collective strategies when a group is stigmatized. This proposition aligns with findings that masking autistic traits were associated with increased internalized stigmatization and discrimination (Botha & Frost, 2020); high autistic identification and open disclosure of one’s diagnosis are reported to be associated with reduced camouflaging strategies (Cage & Troxell-Whitman, 2020). Of note, self-stigmatization may comprise public stigma, including stereotypical beliefs and prejudicial attitudes endorsed by a sizable group in society toward a discredited subgroup (Corrigan & Watson, 2002).

Social camouflaging may result in isolation and social avoidance (Cook et al., 2021), maintaining a widespread misconception regarding autistic people’s preference for solitude and needlessness for social relationships (Maddox & White, 2015). It is suggested that social isolation experienced by many autistic individuals may be mainly due to a lack of interpersonal skills (Maddox & White, 2015) or discrimination experiences (Perry et al., 2021) rather than a lack of desire for relationships. Therefore, a growing body of research reports co-occurrence between autism spectrum and social anxiety disorder (SAD) (Espelöer et al., 2021). SAD is characterized in DSM-5 as “a persistent fear of one or more social or performance situations in which the person is exposed to unfamiliar people or possible scrutiny by others. The individual fears that they will act in a way that will be embarrassing and humiliating” (APA, 2013). SAD is often misdiagnosed for ASD: autistic persons fulfill some of the SAD criteria as they tend to avoid social situations due to fear of negative evaluation and experience physiological symptoms in social situations (e.g., blushing, trembling; Espelöer et al., 2021). However, according to DSM-5 and cognitive-behavioral models (Clark & Wells, 1995), SAD occurs when a person experiences the symptoms excessively and inadequately to the real risk of embarrassment – which in the case of autistic people is debatable as they are at factual risk of discrimination. Spain et al.’s (2018) meta-analysis showed that a handful of studies displayed a significant overlap between social anxiety and low social competencies, including verbal competencies or social context understanding, with people with a diagnosis of SAD (without ASD) showed lower social competences than people with ASD (without SAD). It may suggest that it is the issue of competence, and not the diagnosis itself, that is crucial for fear expression. Therefore, in differential diagnosis, it is fundamental to determine whether the fear is adequate or justified (e.g., due to prior experiences of discrimination; Botha & Frost, 2020), results from a lack of social skills (deriving from autistic traits, not SAD; Spain et al., 2018), or from the fear of social evaluation and self-embarrassment (which would indicate the presence of SAD; Hull et al., 2021b). A scarce body of literature regarding camouflaging and SAD symptoms reports mixed results, as some (Hull et al., 2019, 2021b) point to significant relationships, while others (Shuck et al., 2019; Lai et al., 2017) report contrary.

**Fig. 1:**
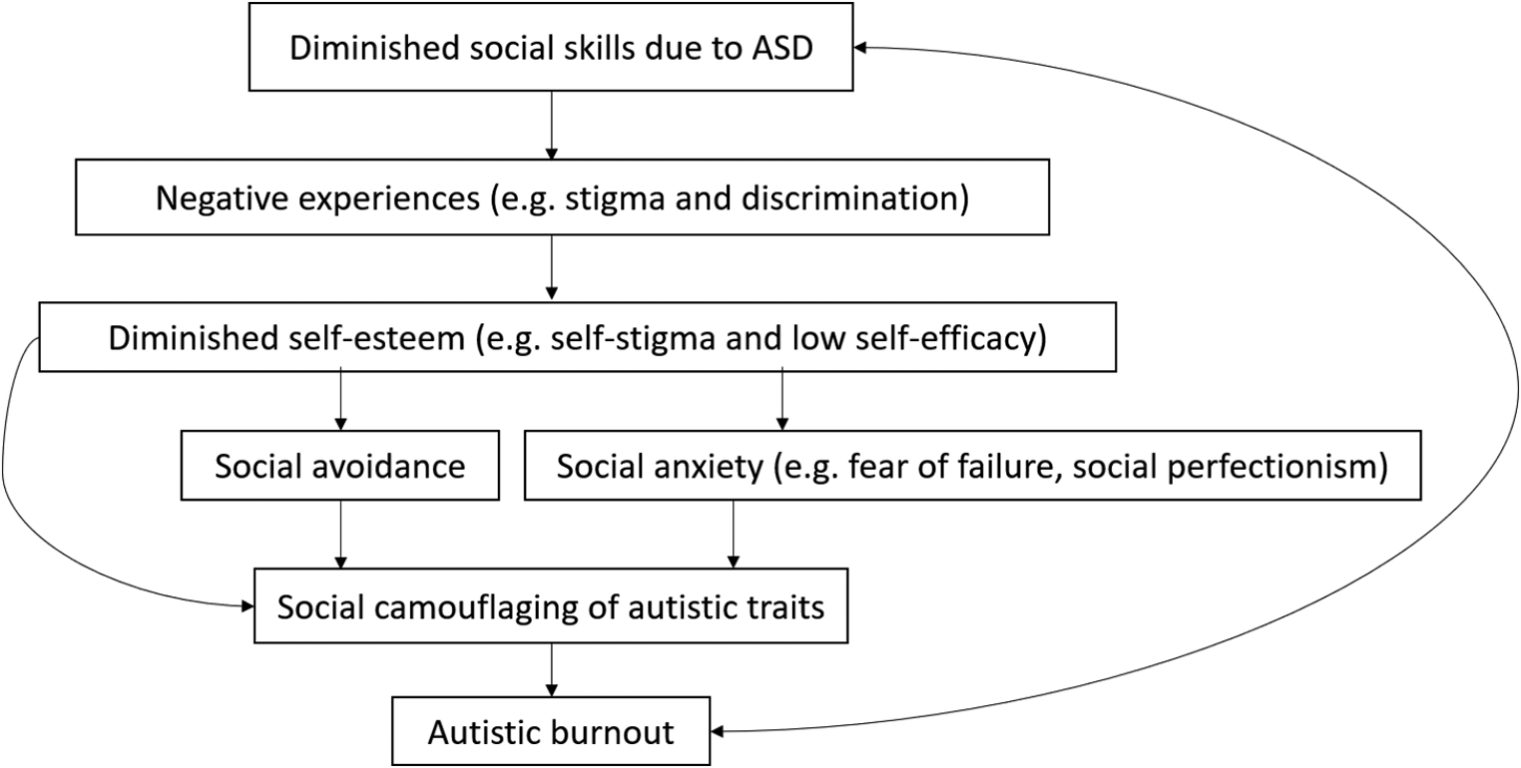
Proposed camouflaging conceptualization

Based on prior literature and clinical research, the focus of the current study was to present a hypothetical conceptualization model of social camouflaging. It is proposed to conceptualize social camouflaging as the behavioral effect of interactions between specific autism spectrum traits, one’s negative social experiences, and self-perception (e.g., negative self-esteem, fear of social failure). Therefore, social camouflaging may derive from negative experiences (including discrimination), diminished social skills (poor theory of mind, reactions considered inadequate by non-autistic peers), and self-esteem (e.g., self-criticism and self-stigma) that lead to social avoidance and social anxiety symptoms (e.g., fear of failure, anxiousness around other people). Notably, social anxiety is associated with social perfectionism, exhibiting inaccurate or impossible to achieve social goals regarding one’s reaction or perception by others (Clark & Wells, 1995). As persons on the autism spectrum display high rates of perfectionism and control demand (Dupuis et al., 2022), this specific aspect of social anxiety may play a significant role in developing a specific need to match with the environment and analyzing what behavior is expected (and rewarded) by others. In turn, avoidance backfires on diminished social skills, while anxiety increases feelings of incompetence and self-criticism. Additionally, social camouflaging, requiring constant social observation and responding in an unintuitive, non-autistic manner, may develop exhaustion and sensory burnout (autistic burnout, Raymaker et al., 2020), causing yet another decline in social skills resulting from overload. In this case, camouflaging would not be described solely as a compensation strategy as it is motivated by negative experiences and self-perception, not by thriving for self-development or a sense of belonging (Perry et al., 2021; Petrolini et al., 2023). A hypothesized model explaining relationships that result in social camouflaging is presented below [Fig. 1].

### The Current Study

The study’s main objective was to explore and conceptualize the psychological mechanisms explaining social camouflaging in persons on the autism spectrum, with a particular focus on social anxiety symptoms. As social camouflaging derives from social discomfort and fear of exclusion (Cook et al., 2021), it was decided to frame an additional, comparative group consisting of individuals with social anxiety disorder diagnosis. Due to common comorbidity (Rosen et al., 2018), a third group of persons with both diagnoses was also devised. Two research goals were formulated. First, to establish differences in social camouflaging features (masking, assimilation, compensation), autistic traits, autistic burnout, social anxiety symptoms (anxiety and avoidance), and social contexts (public stigma) among adult individuals on the autism spectrum, with SAD, or both diagnoses (ASD + SAD). Second, to evaluate associations and determinants of camouflaging based on symptomatology and social contexts, rather than mere diagnosis, (a) a structural equation modeling, and (b) network analysis were designed to shape possible interactions between camouflaging traits and autistic and/or social anxiety factors. As the network approach focuses on studying the dynamics and structure of behavioral systems (Kobourov, 2012), it was hypothesized that it would be more accurate for establishing links between variables in the current study’s question.

## Methods

### Participants

The inclusion criteria for the study were: (1) a declaration of a formal diagnosis of autism spectrum disorder (ASD), or social anxiety disorder (SAD), or both (ASD + SAD); (2) being without an intellectual disability (IQ < 70); (3) being fluent in the Polish language. Due to the high comorbidity of ASD and SAD with other mental health issues (Rosen et al., 2018), the participants were asked to provide additional information in that matter. Persons declaring a self-diagnosis were excluded from the sample. Participants were recruited online through groups and societies aimed at autistic individuals, and stationary mental health facilities in Silesia, Poland (psychiatric and psychotherapeutic offices, outpatient departments). Participants interested in the project filled out an online form via LimeSurvey. The recruitment took place in autumn 2022. All participants provided written informed consent before enrolment in the study. The study was voluntary and unpaid, although all participants were eligible to leave their contact address through a non-affiliated and anonymous link to participate in further paid qualitative studies from this area.

The autism community was involved in the theoretical background of the camouflaging conceptualization, including examples of camouflaging’s effects on exhaustion, social avoidance, and burnout.

Two hundred fifty-four persons participated in the study, including 186 female. One hundred forty-eight participants were on the autism spectrum, declaring having an autism spectrum disorder (ASD) diagnosis, 65 had a SAD diagnosis, and 45 declared ASD + SAD diagnoses. The summary of group characteristics is presented in Table 1.

**Table 1:** Group characteristics

	Autism spectrum (*N* = 148)	Social anxiety disorder (*N* = 65)	Both diagnoses (*N* = 45)
Age (M / SD)	28.97 / 7.44	27.29 / 6.85	28.41 / 6.71
***Gender***			
Female	75.68% (112)	75.00% (48)	57.78% (26)
Male	8.78% (13)	8.78% (7)	17.78% (8)
Non-binary	13.51% (20)	9.37% (6)	20.00% (9)
I would rather not say	2.03% (3)	4.69% (3)	4.44% (2)
***Place of residence***			
Village	8.78% (13)	18.75% (12)	0.00% (0)
City under 50k residents	14.19% (21)	10.94% (7)	15.55% (7)
City over 50k residents	9.46% (14)	14.06% (9)	13.33% (6)
City over 150k residents	18.24% (27)	21.87% (14)	24.44% (11)
City over 500k residents	49.32% (73)	34.37% (22)	46.67% (21)
***Employment status***			
Unemployed	12.16% (18)	23.44% (15)	17.78% (8)
Full-time job	33.11% (49)	32.81% (21)	31.11% (14)
Part-time job	10.81% (16)	4.69% (3)	13.33% (6)
Studying	24.32% (36)	23.44% (15)	22.22% (10)
Working + studying	19.59% (29)	15.63% (10)	15.55% (7)
***Relationship status***			
Single	32.43% (48)	43.75% (28)	35.56% (16)
Informal relationship	41.89% (62)	39.06% (25)	44.44% (20)
Formal relationship	19.59% (29)	14.06% (9)	15.56% (7)
I would rather not say	6.08% (9)	3.13% (2)	4.44% (2)
***Additional diagnosis***			
No	39.86% (59)	35.94% (23)	24.44% (11)
Yes	60.14% (89)	64.06% (41)	75.56% (34)
Types of additional diagnosis/-es *			
Depression	23.65% (35)	32.31% (21)	44.44% (20)
Anxiety disorders **	16.22% (24)	6.15% (4)	17.77% (8)
ADHD	22.97% (34)	21.54% (14)	17.77% (8)
Dyslexia	2.70% (4)	0.00% (0)	0.00% (0)
cPTSD	0.00% (0)	0.00% (0)	2.22% (1)
PTSD	2.70% (4)	1.54% (1)	2.22% (1)
Personality disorders	4.73% (7)	15.38% (10)	6.66% (3)
OCD	3.38% (5)	3.08% (2)	2.22% (1)
Bipolar disorder	3.38% (5)	4.61% (3)	2.22% (1)
Eating disorders	4.05% (6)	3.08% (2)	2.22% (1)
Substance addiction	0.00% (0)	1.54% (1)	0.00% (0)

* some of the participants reported more than one additional diagnosis; ** other than SAD

Due to the sociocultural context and population homogeneity, as the research was conducted in Poland among Polish participants, data on race/ethnicity status was not recorded.

### Measurements

Camouflaging. The Camouflaging Autistic Traits Questionnaire (CAT-Q, Hull et al., 2019) was used. It consists of 25 items (e.g., “In social situations, I feel like I’m “performing” rather than being myself”), rated on a 1 (definitely disagree) to 7 (definitely agree) scale, regarding three subtypes of camouflaging: compensation, masking, assimilation. The reliability rates for the current study were: (a) total score α = 0.70, ώ=0.70, (b) compensation α = 0.81, ώ=0.82, (c) masking α = 0.74, ώ=0.75, (d) assimilation α = 0.81, ώ=0.82.

Autistic traits. The Autism Quotient-10 (AQ-10, Allison et al., 2012) was used. The scale consists of 10 items (“I find it difficult to work out people’s intentions”); in the current study, a four-point response scale was used (Bertrams, 2021). The reliability rates for the current study were: α = 0.68, ώ=0.70, in line with previous works regarding AQ-10 internal reliability and homogeneity (Bertrams, 2021; Taylor et al., 2020).

Autistic burnout. The AASPIRE’s Autistic Burnout Scale (Raymaker et al., unpublished, Polish version by Pyszkowska) was used. The scale consists of 27 items (e.g., “In the past three months, I’ve had a harder time tolerating sensory input than I usually do”), rated from 0 (strongly disagree) to 4 (strongly agree). It has been assessed and used by Arnold et al. (2023) to conceptualize autistic burnout in an adult autistic population. The reliability rates for the current study were: α = 0.95, ώ=0.95.

Social anxiety features. The Liebowitz Social Anxiety Scale (LSAS-SR; Liebowitz, 1987) was used, consisting of examples of 24 social situations (e.g., working while being observed) that are each rated for level of fear from 0 (none) to 3 (severe) and avoidance from 0 (none) to 3 (usually) for the past week. The reliability rates for the current study were: (a) social fear α = 0.86, ώ=0.86, (b) social avoidance α = 0.84, ώ=0.84. The LSAS-SR was previously used in the autistic population, proving satisfactory reliability scores (Bejerot et al., 2014).

Public stigma. The Perceived Public Stigma Scale (PPSS; Chan & Lam, 2017, Polish translation by Pyszkowska, Rożnawski, & Farny, 2021). The Scale contained eight items (e.g., “Most people feel that having an ASD is a sign of personal failure”) adapted from Green’s (2001) study. The items were rated on a six-point Likert scale ranging from 0 (strongly disagree) to 5 (strongly agree). To the author’s knowledge, the scale has not been used in the autistic population before (although it was used in the population of parents of children with ASD, e.g., Pyszkowska et al., 2021; Chan & Lam, 2017). The reliability rates for the current study were: α = 0.70, ώ=0.70.

### Data Analysis

Correlations were conducted using Kendall’s *tau b* (Shober et al., 2018). Analysis of variance (ANOVA) was applied to compare differences between three groups (ASD, SAD, ASD + SAD) regarding variables studied, using mean square and *F* (variation between sample means / variation within the samples) values. Post-hoc Bonferroni corrections were used for significant differences using the bootstrap method for 10,000 samples.

Structural equation modeling (SEM) was designed to establish the significance of models predicting camouflaging and autistic burnout, as mentioned in the conceptualization model above. In this case, only data from persons with ASD or ASD + SAD samples was used. It was decided to perform variance-based SEM (VB-SEM) in partial least squares structural equation modeling (PLS-SEM, Hair et al., 2019). The current study’s design encountered circumstances predisposing to VB-SEM: (1) relatively small sample, (2) exploratory design, (3) correct model specification cannot be ensured (Wong, 2010). PLS-SEM estimates partial model structures by combining principal components analysis with ordinary least squares regressions (Mateos-Aparicio, 2011). Latent variables are composed of items (“indicators”), with the indicator’s satisfactory reliability being > 0.70 or higher (in exploratory research, it is > 0.40, e.g., Hulland, 1999), and convergent validity (average variance extracted, AVE) being higher than 0.50 (Bagozzi & Yi, 1988). Standardized Root Mean Square Residual (SRMR) was the indicator of the model’s fit, with values less than 0.10 or 0.08 (in a more conservative version; Hu & Bentler, 1998) considered a good fit. To assess collinearity issues of the inner model, Variance Inflation Factor (VIF) values were obtained, with VIF values being 5 or lower to avoid the collinearity problem (Hair et al., 2010). Two PLS-SEM models were performed: (1) factors predicting camouflaging with a total score, (2) factors predicting three features of camouflaging: masking, compensation, assimilation. Variables standardization and the bootstrap method with 10,000 samples were applied.

To perform network analysis, signed weighted concentration (partial correlation) networks were created using the Qgraph package of JASP software with an EBICglasso estimator. Regularized Gaussian graphical models (GGMs, cf. Costantini et al., 2015) were used in the analysis, assuming no common latent variables. GGMs focus on the interplay between observable components (the network) and their role in shifting behavior. In this approach, “nodes” are the independent variables, and “edges” are partial correlation coefficients between the variables, indicating conditional dependence (potential causality) between the nodes they connect (Kobourov, 2012). Three nodes were selected for the current study: (1) camouflaging factors (masking, assimilation, compensation), (2) autistic factors (AQ total score, autistic burnout), and (3) social anxiety factors (anxiety, avoidance). Qgraph’s “spring” layout was used to render networks. Thinner edges represented weaker coefficients while thicker – stronger ones (Kobourov, 2012). The bootstrap method for 10,000 samples was also applied.

Calculations were made using the JASP 0.12.2.0 statistical package (University of Amsterdam, Amsterdam, The Netherlands, 2018); for the PLS-SEM, SmartPLS 4.0.8.5. version was used. An α level of 0.05 was considered statistically significant for all statistical tests.

## Results

First, Kendall’s *tau b* correlation analysis was conducted on a total sample. The results are presented in Table 2, including a normality test result (*W* Shapiro-Wilk’s normality test).

**Table 2:** Kendall’s tau b correlation analysis and W normality test

	W	1.	2.	3.	4.	5.	6.	7.	8.
1. CAT-Q_total	0.97***	—							
2. CAT-Q_compensation	0.98**	0.64***	—						
3. CAT-Q_masking	0.98***	0.53***	0.27***	—					
4. CAT-Q_assimilation	0.95***	0.52***	0.26***	0.22***	—				
5. Autistic traits	0.95***	0.16***	0.25***	− 0.12**	0.22***	—			
6. Autistic burnout	0.98**	0.30***	0.22***	0.16***	0.32***	0.13**	—		
7. Social anxiety – anxiety	0.98***	0.29***	0.19***	0.15***	0.38***	0.19***	0.32***	—	
8. Social anxiety – avoidance	0.99	0.30***	0.19***	0.14***	0.37***	0.15***	0.28***	0.65***	—
9. Public stigma	0.98**	0.19***	0.13**	0.10**	0.20***	0.05	0.19***	0.21***	0.20***

** *p* < .01, *** *p* < .001

The total score of camouflaging correlated moderately and significantly with autistic burnout (*tau* = 0.30, *p* < .001), and both factors of social anxiety (anxiety symptoms *tau* = 0.29, *p* < .001, avoidance symptoms *tau* = 0.30, *p* < .001); weak associations were established with autistic traits (*tau* = 0.16, *p* < .001) and public stigma (*tau* = 0.19, *p* < .001). The compensation feature of camouflaging was the strongest correlate of autistic traits (*tau* = 0.25, *p* < .001); the assimilation feature showed moderate associations with both symptoms of social anxiety (anxiety *tau* = 0.38, *p* < .001; avoidance *tau* = 0.37, *p* < .001). Public stigma was associated with assimilation (*tau* = 0.20, *p* < .001) and both features of SAD (anxiety *tau* = 0.21, *p* < .001; avoidance *tau* = 0.20, *p* < .001) but exhibited an insignificant (*p* > .05) relationship with autistic traits.

Then, a comparison of three subgroups was performed using an ANOVA analysis. The results are summarized in Table 3.

**Table 3:** ANOVA test results

	Autism spectrum (*N* = 148)		SAD (*N* = 64)		Both diagnoses (*N* = 45)		df	F	*p*	d	Significant comparisons *
	M	SD	M	SD	M	SD					
1. CAT-Q_total	121.86	20.70	124.11	17.16	122.82	15.67	2	0.862	0.424	0.007	
2. CAT-Q_compensation	43.14	9.42	39.37	9.54	44.49	8.28	2	5.028	0.007	0.038	ASD > SAD; ASD + SAD > SAD
3. CAT-Q_masking	36.26	7.77	39.67	4.57	36.02	6.78	2	5.440	0.005	0.041	SAD > ASD; SAD > ASD + SAD
4. CAT-Q_assimilation	41.45	8.15	44.08	6.86	44.31	6.67	2	4.033	0.019	0.031	SAD > ASD; ASD + SAD > ASD
5. Autistic traits	7.61	1.89	6.27	2.20	8.31	1.86	2	16.379	< 0.001	0.114	ASD > SAD; ASD + SAD > SAD
6. Autistic burnout	61.05	22.82	64.09	18.25	69.73	18.22	2	3.001	0.052	0.023	ASD + SAD > ASD
7. Social anxiety – anxiety	39.22	15.13	46.73	11.99	47.51	13.43	2	9.712	< 0.001	0.071	SAD > ASD; ASD + SAD > ASD
8. Social anxiety – avoidance	36.55	12.68	42.52	12.07	43.89	13.10	2	8.590	< 0.001	0.063	SAD > ASD; ASD + SAD > ASD
9. Public stigma	21.75	7.14	21.83	6.95	23.67	7.77	2	1.289	0.277	0.010	

* significant comparisons are Dunn’s post-hoc results with Bonferroni’s correction

The total score of camouflaging and pubic stigma did not differentiate the subgroups. The compensation feature of camouflaging was significantly higher in persons on the autism spectrum, regardless of SAD. The masking feature was significantly higher among persons with only SAD. The assimilation feature was significantly higher among all persons with SAD, regardless of autism. Autistic traits, assessed by the AQ total score, were significantly higher among persons on the autism spectrum, and even higher for those who also had SAD. Autistic burnout was significantly higher in persons with both autism and SAD when compared to persons with either alone. When compared to autism alone, both social anxiety disorder factors were significantly higher among persons with SAD and highest among those with both SAD and autism.

**Fig. 2:**
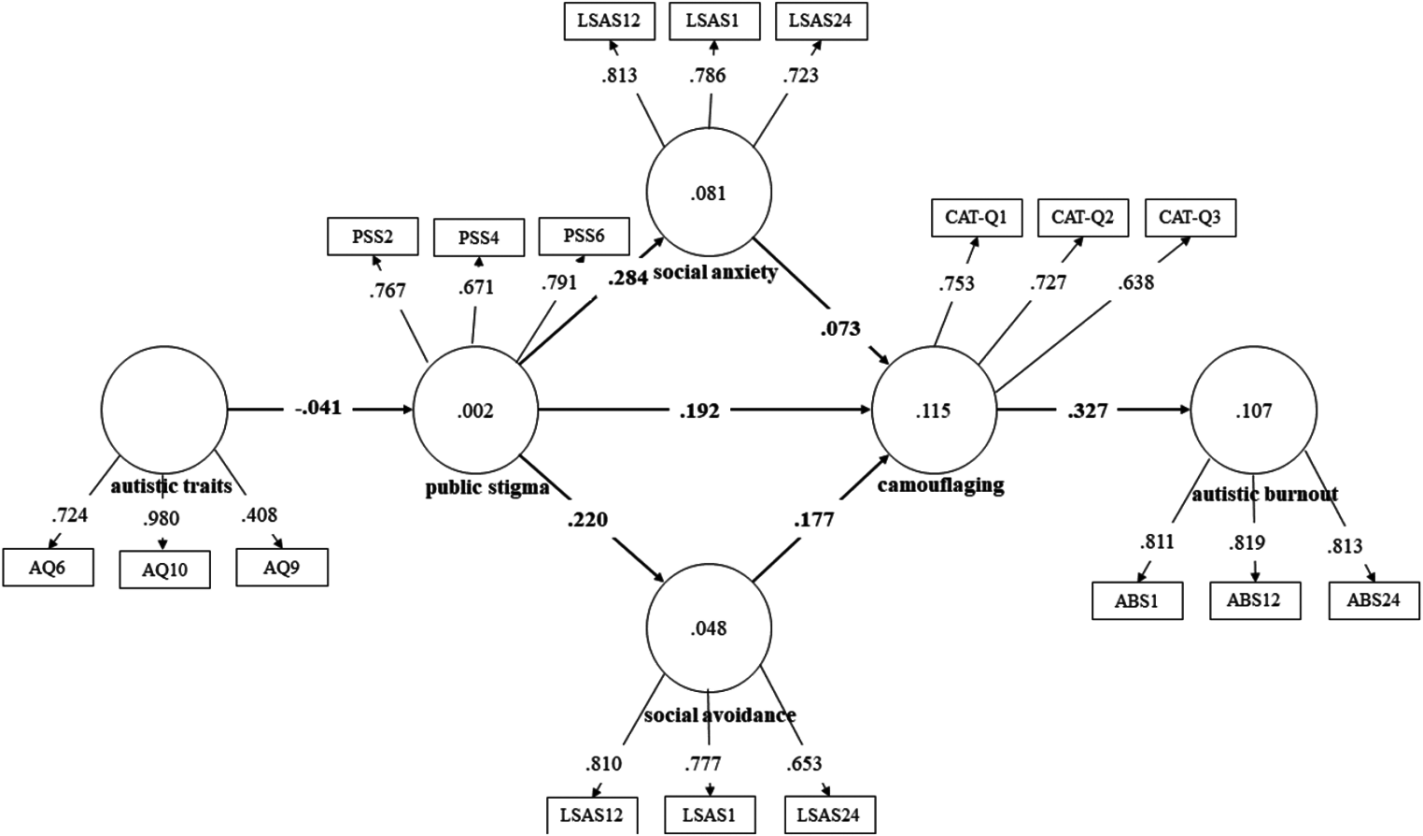
Partial least squares structural equation modeling results for model *a*

**Fig. 3:**
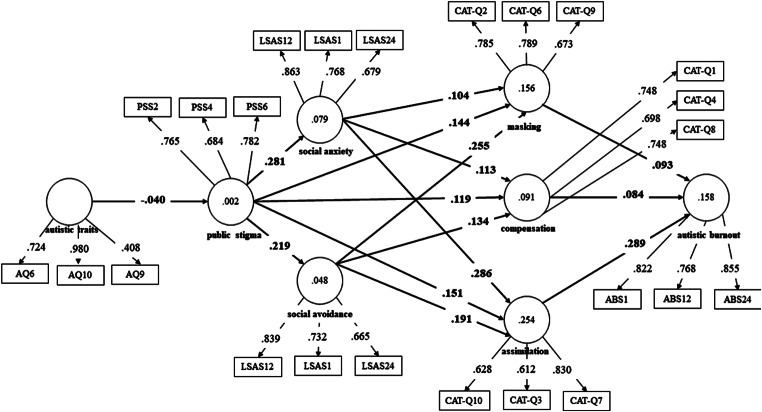
Partial least squares structural equation modeling results for model *b*

In the next step, partial least squares structural equation modeling (PLS-SEM) was applied to verify the fitness of two models predicting camouflaging. The models were designed based on the proposed camouflaging and autistic burnout conceptualization presented in the Introduction section, therefore only ASD and ASD + SAD subgroups were used in this calculation (*N* = 193). In both models, autistic traits, public stigma, social anxiety and social avoidance were predictors of camouflaging and autistic burnout. Model *a* used a total score of CAT-Q, and model *b* used scores of three camouflaging features (masking, assimilation, compensation). Fig. 2 (model *a*) and Fig. 3 (model *b*) show the PLS path modeling estimations.

For model *a*, the results showed that the model tested explained 11.5% of camouflaging total score and 10.7% of autistic burnout. The model showed acceptable fit with Standardized Root Mean Square Residual (SRMR) factor = 0.087. Indicators’ reliability and validity were obtained. Most outer loadings scored higher than 0.70 with five two below (PSS4 = 0.671, CAT-Q3 = 0.638, LSAS21 for social avoidance = 0.653, AQ9 = 0.408), and AVE scores being higher than 0.50. Public stigma predicted both social anxiety (*β* = 0.284, *p* < .05) and social avoidance (*β* = 0.220, *p* < .05). The strongest predictors of camouflaging were public stigma, with *β* = 0.192 (*p* < .05), and social avoidance, with *β* = 0.177 (*p* < .05). Camouflaging showed a substantial effect on autistic burnout (*β* = 0.327, *p* < .05). Autistic traits showed little effect on public stigma, with *β*=-0.041 (*p* > .05).

In model *b*’s case, the results showed that the model tested explained 15.6% of masking, 9.1% of compensation, 25.4% of assimilation, and 15.8% of autistic burnout. The model showed acceptable fit with Standardized Root Mean Square Residual (SRMR) factor = 0.090. Indicators’ reliability and validity were obtained. Most outer loadings scored higher than 0.70 with nine below (AQ6 = 0.408, LSAS24 for social anxiety = 0.679, PSS4 = 0.684, LSAS24 for social avoidance = 0.665, CAT-Q3 = 0.612, CAT-Q4 = 0.698, CAT-Q9 = 0.673, CAT-Q10 = 0.628), and AVE scores being higher than 0.50. Social avoidance most strongly predicted masking (*β* = 0.255, *p* < .05). In contrast, social anxiety showed the highest effect on assimilation (*β* = 0.286, *p* < .05). Public stigma showed similar effects on all camouflaging’s features (*β*s range from 0.119 in compensation to 0.151 in assimilation, *p*s < 0.05). Assimilation showed the strongest effect on autistic burnout (*β* = 0.289, *p* < .05). Similarly to model *a*, autistic traits showed little effect on public stigma, with *β* = − 0.040 (*p* > .05).

**Fig. 4:**
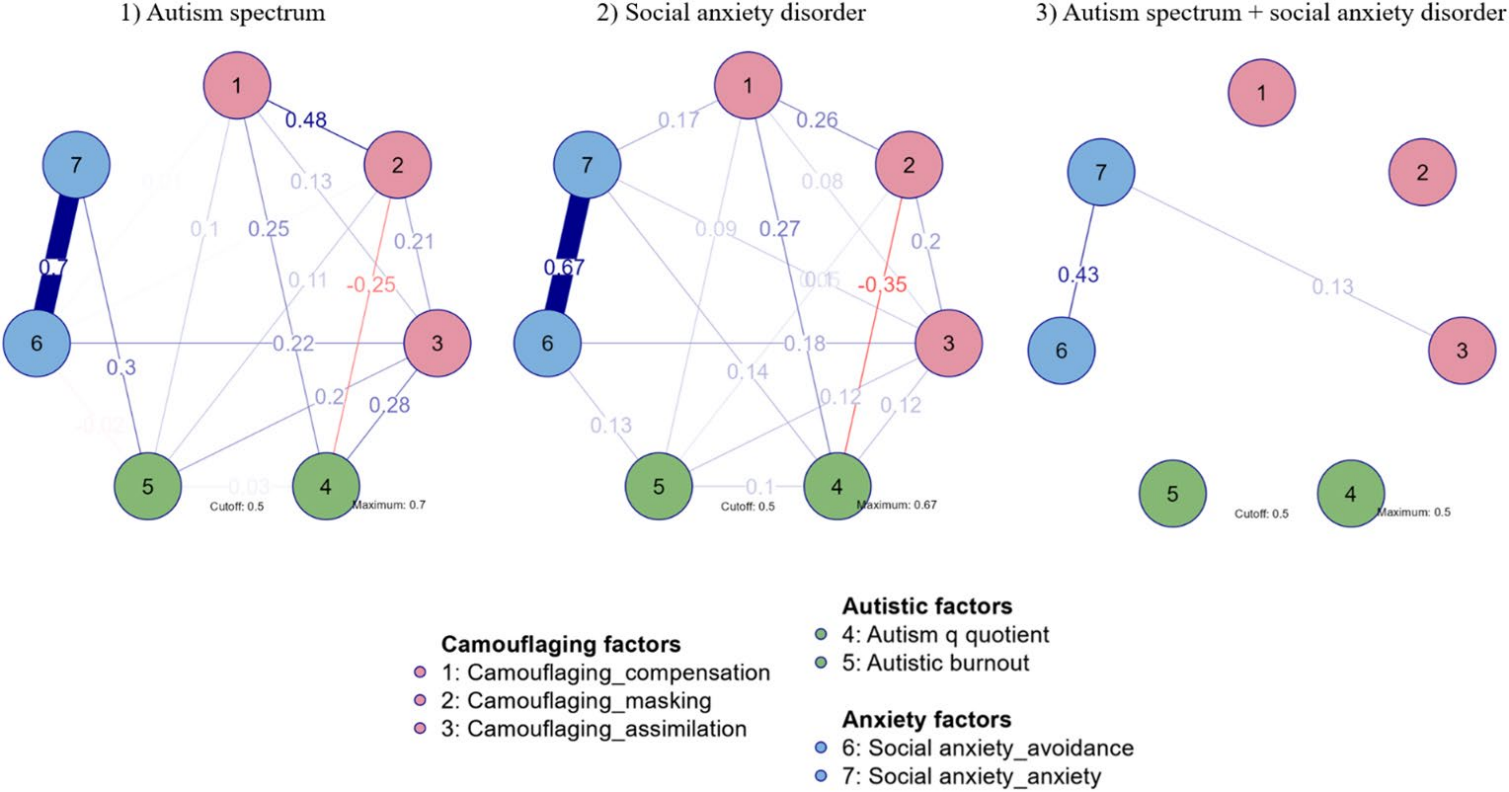
Network plots

**Fig. 5:**
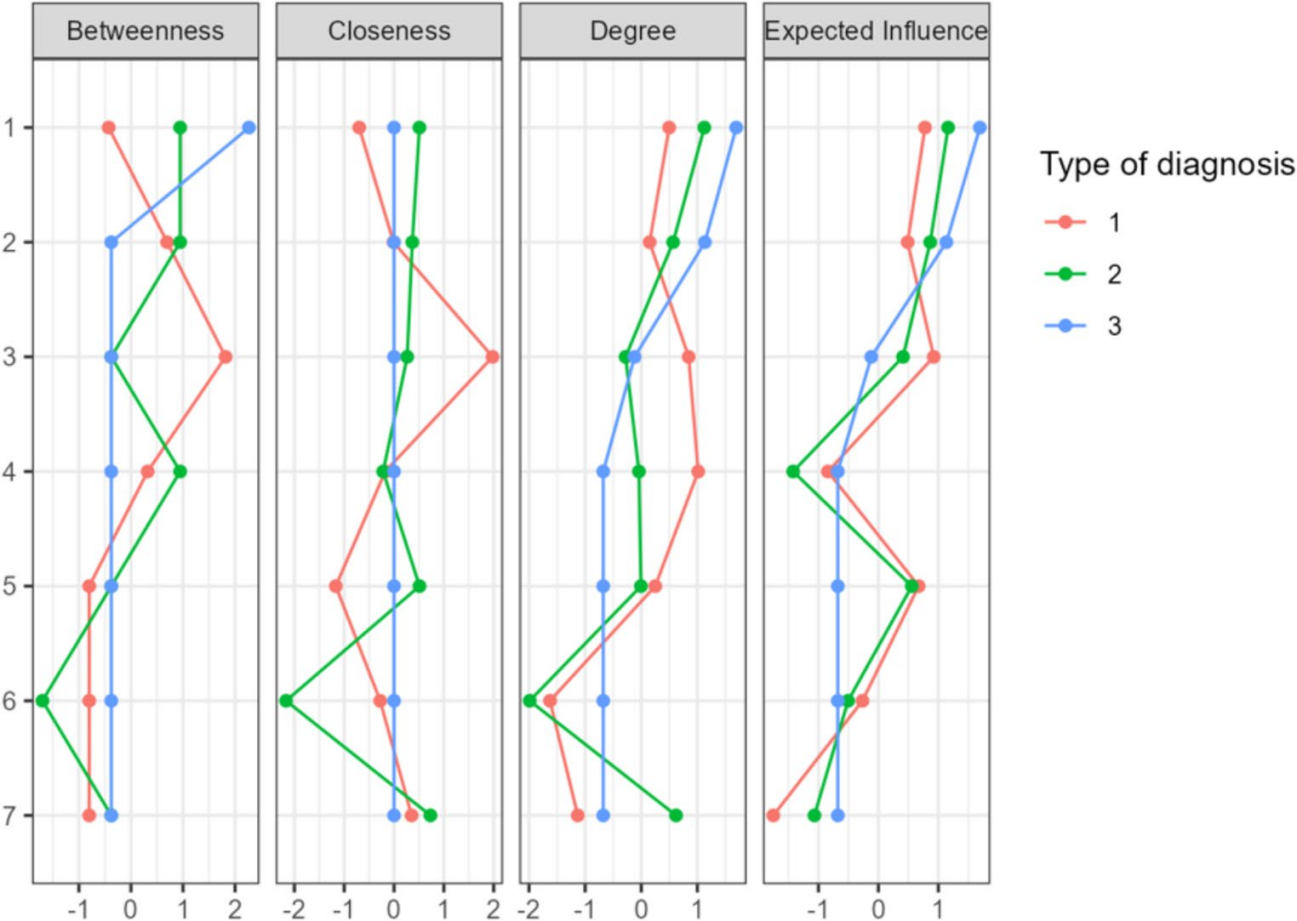
Centrality plots

To outline possible interactions between significant variables (camouflaging, autistic traits, autistic burnout, social anxiety factors), network analyses were applied for three subgroups: (1) autism spectrum, (2) social anxiety disorder, (3) both diagnoses. Pink nodes represented camouflaging factors (1. compensation, 2. masking, 3. assimilation), green nodes autistic factors (4. autistic traits, 5. autistic burnout), and blue nodes – anxiety factors (6. avoidance symptoms, 7. anxiety symptoms). Fig. 4 presents network plots, and Fig. 5 centrality plots for the three groups studied.

In the autism spectrum network, autistic traits scored the highest expected influence (*EI*=-1.753), while in the SAD network, it was the masking feature of camouflaging (*EI*=-1.423); in a network of ASD + SAD’s highest expected influence was affective (anxiety) factor of social anxiety (*EI* = 1.693). The autism spectrum (sparsity = 0.238) and SAD (sparsity = 0.238) networks showed 16 non-zero edges, while the ASD + SAD diagnoses network showed only 2 (sparsity = 0.905). Each of the camouflaging factors was connected with autistic burnout and autistic traits on a similar level (0.25-0.28), with masking presenting a negative relation with autism quotient score (-0.25). No connections with social anxiety affective (anxiety) factor were obtained. In contrast, the social anxiety avoidance factor was related to the assimilation feature of camouflaging (0.22). Similar results were obtained in a SAD network. However, with differences in relations between social anxiety affective (anxiety symptoms) factor being associated with compensation (0.17) and assimilation (0.06), avoidance factor of SAD being related to autistic burnout symptoms (0.13), and affective factor with autistic traits (0.14). In a network consisting of persons with both diagnoses, the only relation was between social anxiety affective (anxiety) factor and assimilation (0.13).

## Discussion

The present study’s objective was to explore and conceptualize links between social camouflaging and social anxiety symptoms in adults on the autism spectrum compared to persons with social anxiety disorders. The total score of camouflaging did not differ between the groups studied (although ASD + SAD sample scored significantly higher in masking and assimilation), suggesting that a tendency to camouflage is rather transdiagnostic, deriving from anxiousness and negative self-perception, not from being autistic per se. Additionally, the findings suggest that autistic burnout and social anxiety symptoms are significant in camouflaging strategies, with autistic traits being of secondary importance. The path analysis exhibited an acceptable fit in the conceptualization model proposed, with autistic burnout being an outcome variable predicted by public stigma, social anxiety symptoms, and camouflaging. These results allow for clinical implications and future research directions, limitations are also discussed.

### Differences Between ASD, SAD, and ASD + SAD Samples

Masking and assimilation features of camouflaging were significantly higher among persons with social anxiety disorder and dual diagnoses (ASD + SAD). It is worth noting that both of these factors are associated with behavioral outcomes and specific strategies (e.g., social observations and appropriate social responses in assimilation, holding back from stimming, etc.). On the other hand, compensation was significantly higher in the ASD group as it is represented by behavioral modifications made by autistic individuals to blend into social situations. These comparative analyses seem to align with Espelöer et al.’s (2021) research reporting that social anxiety symptoms in individuals with ASD are primarily based on deficits in social competence, and the symptoms associated with both diagnoses run like a vicious circle. In this light, another finding in the current study seems understandable, as persons with ASD + SAD scored significantly highest in social anxiety symptoms and autistic traits.

Although it was marginally significant (*p* = .052), autistic burnout was highest in persons with ASD + SAD, acting as a constant predictor and correlate of camouflaging, showing significance in relationships with all its features. Interestingly, it was not associated with the AQ score through network analysis in the ASD sample (with weak association in the SAD sample), although it was linked with camouflaging and anxiety (in an ASD sample) and avoidance (in the SAD sample) symptoms. Therefore, these results support a hypothesis that the burnout experienced by persons in the ASD population may be due to personal and environmental demands (including stigma and discrimination), diminished well-being, and mental strain (including anxiousness and distress), being in accordance with Mantzalas et al.’s (2022) conceptual model for autistic burnout. In that model, “personal demands” include masking or camouflaging, sensory sensitiveness, and autistic traits – all factors were interrelated in the current study, proving that these dynamics may affect each other. Additionally, these results suggest that a measurement of “autistic burnout” (e.g., AASPIRE’s Autistic Burnout Scale used in this study) may be, in fact, a measure of sensory or cognitive overload that may appear in different contexts and psychopathologies (e.g., SAD). Therefore, further studies should focus on more specific aspects of the “autistic” aspects of burnout experienced in the result of social exhaustion (Arnold et al., 2023).

### Conceptualization Model Verification

Structural equation models showed that the proposed conceptualization, with camouflaging and autistic burnout as the outcome variables, exhibited an acceptable fit. However, it must be highlighted that it was on the verge of acceptability (0.09-0.08, cf. Hu & Bentler 1998). Autistic traits exhibited minimal prediction of public stigma (less than 1%), yet public stigma predicted 8% of social anxiety and 4% of social avoidance. The effects of public stigma and SAD features varied in terms of the camouflaging total score and individual camouflaging features: assimilation was predicted most strongly (25%) and compensation least strongly (9%) by the model tested, with a combined CAT-Q score being predicted by 11%. These results suggest that camouflaging should be considered a complex behavioral strategy with a specific function of its features, consistent with previous works by Jorgenson et al. (2020) or Bernardin et al. (2021). Furthermore, in both models tested, camouflaging resulted significantly in autistic burnout, implying that this strategy (or *each* of the strategies presented in camouflaging) is costly and may result in exhaustion. The models did not point to the importance of autistic traits in either camouflaging or public stigma, which – according to previous research (e.g. Botha & Frost, 2020; Perry et al., 2021) – shows that negative self-perception and strategies to “pass” as non-autistic most significantly derives from environmental factors, not “autistic traits”.

In line with previous works, a masking feature of camouflaging exhibited negative associations with autistic traits (Wiskerke et al., 2018). As masking is related to the suppression of autism-related behaviors (e.g., motor stereotypies) and/or intentionally exhibiting neurotypical behaviors (e.g., maintaining eye contact), persons who use masking may appear as “non-autistic”. Of note, masking was positively associated with autistic burnout, highlighting that it requires cognitive and behavioral effort, resulting in exhaustion. No anxiety symptoms were significant in relationships with masking, perhaps due to being related to social demands of “passing” as non-autistic and coming as a natural social reaction for many autistic individuals (Raymaker et al., 2020; Hull et al., 2017). Further research in this area is needed. With autistic traits showing the highest negative expected influence in the ASD sample, it can be hypothesized that the dynamics between camouflaging, social anxiety, and autistic burnout vary in terms of autistic symptoms’ intensification – as proposed by Petrolini et al. (2023) or Kapp et al. (2019) who highlight that the engagement in camouflaging strategies may be affected by one’s social skills or executive functions.

Assimilation was the feature strongest predicted by social anxiety. These results may imply that assimilation – understood as putting one’s effort into fitting in and not being recognized as “different” (Hull et al., 2019) – derives from the fear of standing out and behaviors obtained in this strategy may be used to “pass” as a neurotypical (Perry et al., 2021). However, while actions such as forcing oneself to talk to a stranger or attending a crowded party enable one to pass as non-autistic (or less autistic) and partially improve social interactions, it can be dissonant and unauthentic (ego-dystonic) and motivated by fear (of rejection, stigmatization, etc.), not harmonious to one’s values and needs (ego-syntonic). Therefore, it can be sensory and cognitively exhausting, and – according to prior research in line with the current study – results in diminished well-being and overload (Cook et al., 2021; Raymaker et al., 2020). However, it should be noted that these outcomes do not contradict the fact that autistic individuals long for connection and sense of belonging, engaging in socializing and assimilating to their social environment (Cage & Troxell-Whitman, 2020) – even if it may result in overstimulation or masking.

The results partially confirm the assumptions regarding the relationships between social camouflaging, public stigma, social anxiety symptoms, and autistic burnout. Consistently with a hypothesized conceptualization, camouflaging exhibited higher associations with autistic burnout than autistic traits. Similarly, a total camouflaging score correlated more with social anxiety symptoms (fear and avoidance) and public stigma than the AQ-10 total score. Public stigma displayed no significance in relationship with autistic traits. However, it was correlated with autistic burnout, all features of camouflaging, and both aspects of SAD symptoms being of particular importance in predicting assimilation camouflaging. Additionally, the total score of camouflaging did not differ between the groups studied, while masking and assimilation tendencies were higher among persons with a SAD diagnosis, with compensation being significantly higher among individuals with the ASD diagnosis. These results may suggest that the presence of the autistic traits (or ASD diagnosis) may be less relevant for engaging in a camouflaging strategy and result from a combination of sensory overload, social anxiety symptoms, and negative self-concept (Raymaker et al., 2020; Hull et al., 2021b), not being a part of the autistic phenotype itself. Further research regarding camouflaging should be conducted on various non-autistic populations to compare these strategies.

### Limitations

Despite its strengths, the current study had its limitations. First, due to the study’s exploratory nature and cross-sectional design, the results cannot be generalized, and the hypothesized camouflaging conceptualization cannot be approved or declined based on the current research. However, the outcomes seem promising and prepare the ground for further examinations, especially in the experimental design. Second, the sample was unbalanced and homogenous, comprised of mostly white, employed or student-enrolled adults living in Poland, dominated by females. Although camouflaging appears more often in women (Cook et al., 2021), it seems necessary to further understand male and non-binary perspectives as they seem highly underrepresented in the current camouflaging literature. The sample’s diagnoses were also unbalanced, with a dominance of the ASD group. The population studied experienced public stigma in a Polish context (e.g., low social awareness about the autism spectrum or mental health; common beliefs that one cannot stand out; diminished tolerance for any differences, cf. Pisula et al., 2024) and further research needs to focus on how it manifests in different countries or cultures. However, it needs to be highlighted that vast majority of previous research in this area was conducted in the Anglo-Saxon context hence the current study significantly adds to the existing knowledge in this area. Another issue that needs to be addressed is the small number of measurements aimed at different symptoms or factors of ASD/SAD, which disallows for a deeper understanding of specific aspects significant for developing camouflaging strategies (e.g., empathizing vs. systemizing, social perfectionism, fear of failure, etc.). In that context, the use of the AQ-10, a standard measure of autistic traits in research, was understandable but posed difficulties in terms of unsatisfactory reliability rates (α = 0.68, ώ=0.70), which was in line with previous works regarding internal reliability and homogeneity issues (Bertrams, 2021). Additionally, AQ-10 was used in all three samples, with the average score of each group was more than 6 (in a SAD sample it was M = 6.27) indicating that the cut-off point of the scale was exceeded. It is consistent with Taylor et al.’s (2020) work that highlighted that AQ-10 is not a psychometrically robust measure when used in non-autistic samples.

### Clinical Implications and Future Research Directions

The abovementioned findings and limitations allow for distinguishing clinical implications and future research directions. Given that camouflaging was exhibited by all samples studied, this issue should be addressed in working with persons experiencing social anxiety or other social difficulties. Additionally, a context of camouflaging strategy used by autistic individuals should be conceptualized by mental health professionals: whether it is driven by anxiety and fear of rejection or stigma, compensation for social skills, or a way to achieve one’s social or professional goals. Based on that context camouflaging can be assessed as beneficial or maintaining one’s suffering. Furthermore, psycho-education regarding potential advantages (e.g., assimilating to environment, social acceptance) as well as costs (e.g., burnout, risks of developing negative self-image, including self-stigma) of camouflaging should be discussed while working with autistic individuals. In sum, the context and function of camouflaging should always be defined, especially that it can vary in different circumstances and fulfill various needs (or cause symptoms and distress to persist). Considering that autistic burnout was an outcome of camouflaging, additional interventions aimed at emotional regulation and sensory overload-related self-care should be applied (e.g., dialectical-behavioral therapy, DBT, Ritschel et al., 2022).

As camouflaging is a multifaceted behavioral strategy, it must be observed through experimental, not only cross-sectional, lenses: an additional research area should be addressed, focusing on therapeutic approaches to develop self-acceptance, social skills, self-regulation, and sensory overload-related regulation. In turn, further observations should be focused on whether these abilities would affect camouflaging strategies in both ASD and SAD populations. Additionally, as camouflaging is mainly associated with females, it should be considered to develop a further understanding of males’ and non-binary functioning in this area. As the total score of camouflaging did not differ between the ASD and SAD samples, it is suggested to broaden the focus on this strategy among persons with social anxiety. Lastly, as the concept of “camouflaging” is not heterogenous and its features vary in terms of dynamics with different psychological variables, future research should aim at specific aspects of camouflaging to verify the pros and cons of applying this strategy among individuals with difficulties with social interactions.

## Declarations

### Community Involvement

The author consulted the theoretical background of the camouflaging and aims of the study at the autism society group at the University of Silesia in Katowice.
